# Syphilis in Maria Salviati (1499–1543), Wife of Giovanni de’ Medici of the Black Bands

**DOI:** 10.3201/eid2606.AC2606

**Published:** 2020-06

**Keywords:** syphilis, renaissance, Florence, Italy, Medici, sexuality, bacteria, treponematosis, sexually transmitted infections, Maria Salviati

**Figure:**
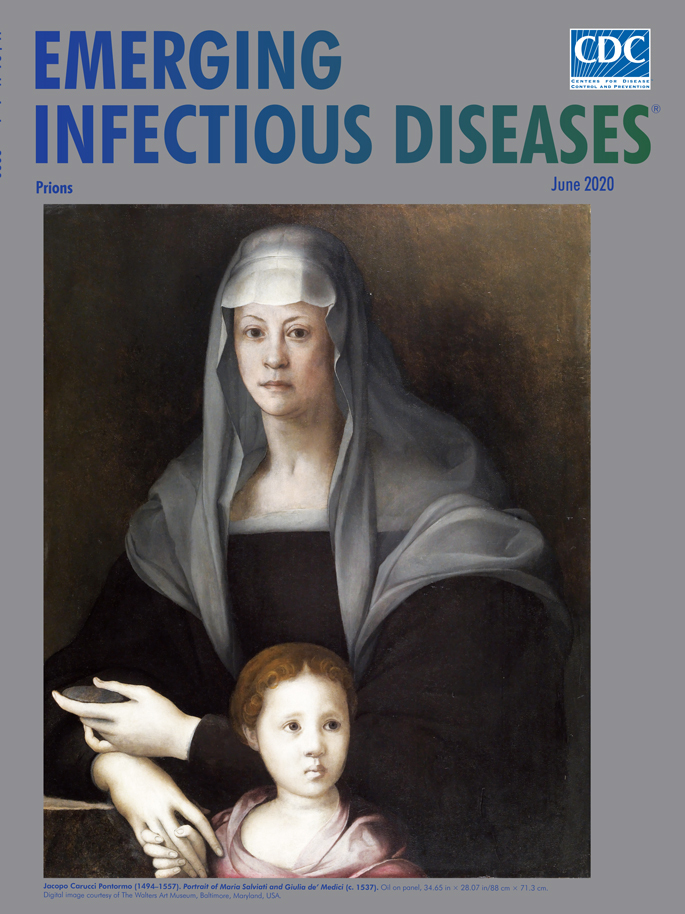
**Jacopo Carucci Pontormo (1494–1557). Portrait of Maria Salviati and Giulia de’ Medici (c. 1537).** Oil on panel, 34.65 in × 28.07 in/88 cm × 71.3 cm. Digital image courtesy of The Walters Art Museum, Baltimore, Maryland, USA.

## Notice to Readers

Readers may learn more about this month’s cover image, *Portrait of Maria Salviati and Giulia de’ Medici*, by reading the historical review article Syphilis in Maria Salviati (1499–1543), Wife of Giovanni de’ Medici of the Black Bands, which appears in this issue. This image is also included as Figure 1 in that article.

